# Predictive performance of kinetic eGFR, midregional proadrenomedullin, and H3.1 nucleosomes for acute kidney disease in sepsis: a secondary analysis of a large multicentre randomized controlled trial

**DOI:** 10.1186/s40560-026-00926-y

**Published:** 2026-08-19

**Authors:** Caroline Neumann, Frank Bloos, Philipp Franken, Thomas Lehmann, Holger Bogatsch, Johannes Ruhe, The Sepnet Critical Care Trials Group

**Affiliations:** 1 Department of Anesthesiology and Intensive Care Medicine, Jena University Hospital, Am Klinikum 1, 07747 Jena, Germany; 2 Center for Sepsis Control & Care (CSCC), Jena University Hospital, Am Klinikum 1, 07747 Jena, Germany; 3 Center for Clinical Studies, Jena University Hospital, Jena, Germany; 4 Institute for Medical Informatics, Statistics and Epidemiology (IMISE) and Clinical Trial Centre Leipzig, University of Leipzig, Härtelstraße 16-18, 04107 Leipzig, Germany; 5 Department of Internal Medicine III, Nephrology, University Hospital Jena - Friedrich Schiller University, Jena, Germany

**Keywords:** Sepsis, Acute kidney disease, Kinetic eGFR, H3.1 nucleosomes, Midregional pro-adrenomedullin

## Abstract

**Background:**

Sepsis-associated acute kidney injury (SA-AKI) frequently progresses to acute kidney disease (AKD) and is linked to poor outcomes. We evaluated whether combining biomarkers reflecting complementary pathophysiological domains—histone H3.1 nucleosomes (cellular injury), midregional pro-adrenomedullin (MR-proADM; endothelial dysfunction), and kinetic estimated glomerular filtration rate (kinetic eGFR; dynamic renal function)—improves prediction of AKD and renal recovery.

**Methods:**

This secondary analysis of the multicentre randomized SISPCT trial included 690 patients with sepsis after exclusion of patients with pre-existing renal replacement therapy or missing AKI data. Biomarkers and kinetic eGFR were assessed at baseline, day 2, and day 7. Multivariable logistic regression models adjusted for age, sex, and non-renal SOFA score were used to assess associations with AKD. Predictive performance was evaluated using receiver operating characteristic (ROC) curves and area under the curve (AUC). Calibration for the combined models for each day was evaluated using bootstrap-corrected calibration plots. Clinical utility was assessed using decision curve analysis with fivefold cross-validation.

**Results:**

AKD occurred in 196 patients (28.4%). At baseline, only MR-proADM was independently associated with AKD (adjusted OR 1.17, 95% CI 1.08–1.28; *p* < 0.001), whereas H3.1 and kinetic eGFR were not. At day 2 and day 7, only changes in kinetic eGFR were independently associated with AKD (both *p* < 0.001). Discriminative performance increased over time, with AUCs for the combined model of 0.65 at baseline, 0.70 at day 2, and 0.76 at day 7. At later time points, kinetic eGFR consistently showed the highest discriminative performance based on the point estimates of the AUC. An overall good calibration performance was shown. Decision curve analysis demonstrated only modest and inconsistent additional clinical net benefit of the combined model compared with kinetic eGFR alone. AKD was associated with increased 90-day mortality (55% vs. 24%; risk ratio 2.3, 95% CI 1.9–2.9). Biomarkers showed limited and inconsistent performance for prediction of renal recovery.

**Conclusions:**

In patients with sepsis, MR-proADM at baseline and dynamic changes in kinetic eGFR during the first week were independently associated with AKD, whereas histone H3.1 nucleosomes provided limited predictive value. Although multimarker models seemed to modestly improve baseline discrimination, this advantage was not sustained over time and added little clinical benefit beyond kinetic eGFR. These findings highlight the value of dynamic renal function assessment for risk stratification of SA-AKI progression to AKD.

*Trial registration* ClinicalTrials.gov Identifier: NCT00832039.

**Supplementary Information:**

The online version contains supplementary material available at 10.1186/s40560-026-00926-y.

## Introduction

Sepsis-associated acute kidney injury (SA-AKI) is a common and severe complication in critically ill patients, arising from a complex interplay of microcirculatory dysfunction, dysregulated inflammation, endothelial injury, metabolic reprogramming, and thromboinflammation [1]. Despite advances in understanding its multifactorial pathophysiology, early recognition remains challenging, and management is largely supportive, as targeted therapies are still under investigation [2]. Consequently, progression to acute kidney disease (AKD) and impaired renal recovery is frequent, highlighting the need for improved risk stratification. This unmet need for earlier and more accurate risk stratification has driven growing interest in biomarkers that capture complementary dimensions of SA-AKI pathophysiology and may improve prediction of AKD progression and renal recovery.

Histones, particularly H3.1-containing nucleosomes, are released during cell death or neutrophil extracellular trap formation and exert cytotoxic effects on renal endothelium and tubular cells [3]. Experimental work demonstrates that histone exposure induces leukocyte recruitment, microvascular leakage, renal inflammation, and structural features of AKI [4]. In animal models of sepsis, rising histone H3.1 levels parallel worsening tubular injury and renal dysfunction [5] while in human sepsis studies, elevated circulating histones are associated with multiple organ failure and fatal outcome [6]. Clinical studies link elevated circulating H3.1 with tissue injury in infection, inflammation and organ failure [7].

Midregional pro-adrenomedullin (MR-proADM) is a stable surrogate of adrenomedullin reflecting endothelial dysfunction, microcirculatory failure, and systemic inflammation [8]. In critically ill patients, including those with sepsis and AKI after cardiac surgery [9], elevated MR-proADM correlates with disease severity, kidney dysfunction, giving prognostic information for renal replacement therapy (RRT) requirement. Serial measurements provide dynamic prognostic information, as rising levels are associated with ongoing organ failure and death [10, 11] whereas decreasing levels reflect improved microcirculatory function and recovery [10]. However, both markers are not kidney-specific and may be influenced by systemic inflammatory and cardiovascular processes, underscoring the need to interpret them in conjunction with more specific markers.

Kinetic estimated glomerular filtration rate (kinetic eGFR) provides a real-time assessment of renal function during non-steady-state conditions, incorporating changes in serum creatinine (SCr) over time. Unlike conventional eGFR, kinetic eGFR detects AKI earlier and predicts persistent renal dysfunction, need for RRT, and renal recovery with strong prognostic accuracy. Its predictive accuracy for renal recovery has been shown to outperform several novel biomarkers, highlighting its clinical utility in assessing real-time renal function during AKI and recovery [12, 13].

Integrating H3.1, MR-proADM, and kinetic eGFR as complementary domains—cellular injury, endothelial/microcirculatory dysfunction, and dynamic renal function—may ameliorate the early identification of patients at risk for AKD and impaired renal recovery. This study evaluated the predictive performance of these biomarkers, individually and in combination, in a large cohort of patients with sepsis admitted to the intensive care unit.

## Material and methods

### Study design and population

This study is a secondary analysis of the multicentre, randomized, controlled SISPCT trial (ClinicalTrials.gov: NCT00832039), which evaluated high-dose sodium selenite and procalcitonin-guided antibiotic therapy in patients with sepsis and septic shock [14]. Adult patients were enrolled within 24 h of sepsis onset. Patients with immunosuppression, infections requiring prolonged antibiotic therapy, or without a commitment to full treatment were excluded from the original trial.

For the present analysis, patients with pre-existing RRT at baseline were excluded. The study was conducted in accordance with the Declaration of Helsinki and Good Clinical Practice and was approved by the local ethics committees. Written informed consent was obtained from all patients or their legal representatives.

### Definitions and endpoints

Baseline SCr was defined as the lowest value within 24 h prior to study inclusion. Estimated glomerular filtration rate (eGFR) was calculated using the 2021 CKD-EPI equation without the race coefficient [15].

Acute kidney injury (AKI) was defined according to KDIGO criteria based on changes in SCr and/or urine output, or initiation of RRT [16].

Acute kidney disease (AKD) was defined according to ADQI as persistent kidney dysfunction (SCr > 1.5 × baseline or RRT requirement) occurring between day 7 and day 90 [17] after sepsis onset.

The most recent available SCr measurement within the respective time window was used for classification.

Renal recovery (after AKD) was categorized as (a) complete recovery: last observed SCr < baseline and RRT-free for the last 10 days; (b) good partial recovery: improvement with last observed SCr ≤ 1.5 times baseline and RRT-free for the last 10 days; (c) limited partial recovery: improvement with last observed SCr < maximum observed SCr and RRT-free for the last 10 days or (d) no recovery: persistent renal dysfunction (SCr ≥ maximum observed SCr until D21), ongoing RRT dependence or death. For analysis of renal recovery as dichotomic secondary endpoint (a) and (b) versus (c) and (d) were summarized.

The primary endpoint was the occurrence of AKD. Secondary endpoints included mortality and renal recovery.

### Measurement of biomarkers and calculation of kinetic eGFR

Blood samples were collected daily until day 10 and stored at −80 °C in the Integrated Biobank Jena. H3.1 nucleosomes were measured in citrate plasma, and midregional pro-adrenomedullin (MR-proADM) in EDTA plasma at days 0, 2, and 7 using validated assays (details in supplement). Kinetic eGFR was calculated using the modified Chen formula proposed by Dewitte and colleagues [12], which accounts for dynamic changes in SCr during non-steady-state conditions. All laboratory analyses were performed blinded to clinical data.

### Statistical analysis

Continuous variables are presented as mean ± standard deviation or median (interquartile range), as appropriate, and categorical variables as counts and percentages. Group comparisons for baseline values were performed using Student’s t-test, Wilcoxon rank-sum test, Chi-square test, or Fisher’s exact test, as appropriate. Due to their exploratory nature, these analyses were conducted without applying an alpha correction.

Multivariable logistic regression models were constructed to assess the association between biomarkers (H3.1, MR-proADM, and kinetic eGFR) and the occurrence of acute kidney disease (AKD). Separate models were developed for each time point (baseline, day 2, and day 7), incorporating either absolute biomarker values at baseline or changes over time (Δ day *x* value—baseline value) at follow-up time points.

Variables were selected a priori based on clinical relevance and prior literature. The primary variables of interest—kinetic estimated glomerular filtration rate (kinetic eGFR), midregional pro-adrenomedullin (MR-proADM), and H3.1 nucleosomes—were included in all models. Adjustment variables were predefined and included age, sex, and non-renal Sequential Organ Failure Assessment (SOFA) score as established risk factors for adverse renal outcomes in sepsis. No automated variable selection procedures were applied. To reduce the risk of overfitting, the number of covariates was limited relative to the number of outcome events. Continuous variables were analysed on their original scale; H3.1 nucleosome levels were log-transformed due to right-skewed distribution. Collinearity between variables was assessed using variance inflation factors, and no relevant multicollinearity was observed. A two-sided *p*-value < 0.05 in Wald test was considered statistically significant in this primary analysis. For all models, odds ratios (ORs) with corresponding 95% confidence intervals (CIs) were reported. In addition, unadjusted ORs derived from univariable logistic regression analyses were calculated and presented for comparison.

Predictive performance was evaluated using receiver operating characteristic (ROC) curves and corresponding areas under the curve (AUCs), and their 95% confidence intervals (95% CIs). ROC analyses were based on predicted probabilities derived from univariable logistic regression models and from the unadjusted multivariable model including all three predictors. Comparisons were restricted to patients with complete biomarker data to ensure consistency across models. Optimal cut-off values were determined using the Youden index. In addition, sensitivity, specificity, negative predictive value (NPV), positive predictive value (PPV), as well as positive and negative diagnostic likelihood ratios were calculated and reported.

Calibration for the combined models for each day was evaluated using bootstrap-corrected calibration plots (1000 resamples), bootstrap optimism-corrected calibration intercepts and slopes, and Brier score.

Decision curve analysis (DCA) was also performed to evaluate the clinical utility of those models. It was assessed using fivefold cross-validation implemented with the cv_decision_curve() function of the rmda (version 1.6) package in R. For each fold, models were trained on four-fifths of the data and evaluated on the remaining one-fifth. Cross-validated predicted probabilities from all five test folds were then combined to calculate the net benefit across threshold probabilities of 0.1, 0.2, and 0.3. Analyses were performed separately for each time point. Results were visualized by plotting the curves for each model, allowing comparison of their clinical net benefit across the selected threshold probabilities. Model performance was also compared against two default strategies: “All” (assuming all patients are at risk of AKD) and “None” (no risk-based intervention).

ROC analyses were also conducted equivalently for the secondary endpoints 90-day mortality (including all 1089 patients) and renal recovery in the supplement. Missing data were handled using a complete case approach. For each analysis, only patients with available data for all included variables were considered. The proportion of missing data for the primary variables of interest was low; therefore, no imputation was performed.

Patients receiving renal replacement therapy (RRT) prior to or at the time of biomarker assessment were excluded from regression and ROC analyses, as RRT alters creatinine kinetics and circulating biomarker concentrations, precluding reliable estimation of intrinsic renal function and biomarker–outcome associations. All statistical analyses were performed using R (version 4.5.1). For procedures involving random resampling (fivefold cross-validation and bootstrap calibration), a fixed random seed was used to ensure reproducibility.

## Results

### Study population

Of 1089 patients enrolled in SISPCT trial, 118 with pre-existing renal replacement therapy and 281 lacking the data required to determine AKI were excluded, leaving 690 patients for analysis.

**Fig. 1:**
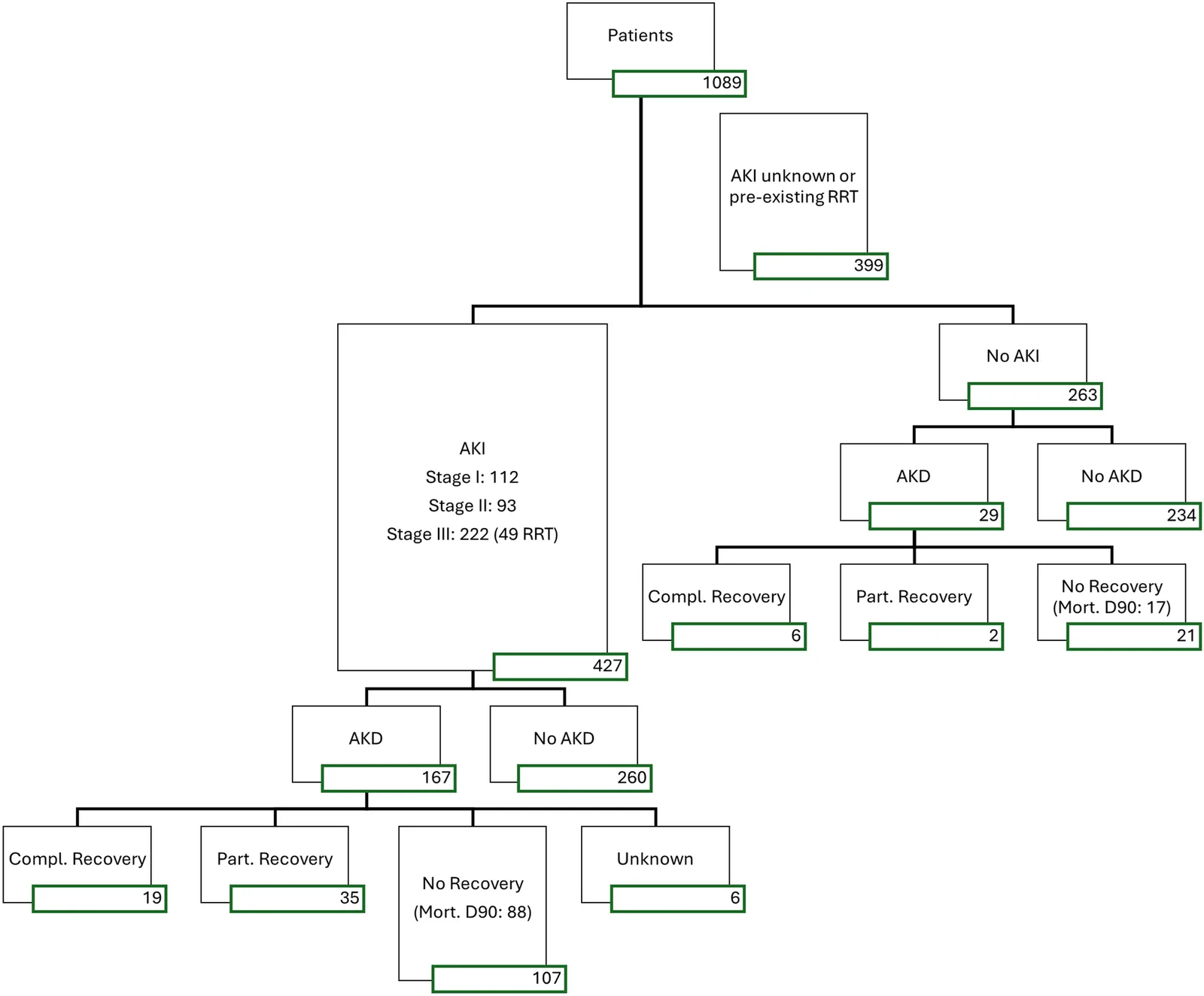
Consort diagram, *AKI* acute kidney injury, *RRT* renal replacement therapy, *part.* partial, *mort.* mortality, *AKD* acute kidney disease, *compl.* complete

Among the included patients, 427 (61.9%) developed AKI during the first 7 days (KDIGO stage 1: *n* = 112 [26.2%], stage 2: *n* = 93 [21.8%], stage 3: *n* = 222 [52.0%]). AKD developed in 167 of these patients (39.1%). An additional 29 patients fulfilled AKD criteria without preceding AKI, resulting in 196 patients (28.4%) with AKD overall (Fig. 1, consort diagram).

Baseline characteristics of the cohort including demographics, comorbidities, and disease severity are described in detail in Table 1. Compared with patients without AKD, those developing AKD were older, more frequently male, had higher illness severity scores, lower kinetic eGFR, and higher circulating MR-proADM and H3.1 concentrations at study inclusion.

**Table 1:** Baseline characteristics of the study cohort

Variable	Total *N* = 690	AKD *N* = 196	No AKD *N *= 494
Female sex, n (%)	232 (34)	52 (27)	180 (36)
Age, mean (SD), years	66.1 (13.2)	67.8 (11.2)	65.4 (13.9)
Comorbidities, *n* (%)			
DM	168 (24)	49 (25)	119 (24)
Liver cirrhosis	52 (7.5)	19 (9.7)	33 (6.7)
Severity scores, mean (SD)			
SOFA, points	10.2 (3.2)	11.4 (3.4)	9.8 (3.0)
SOFA without renal, points	8.8 (2.7)	9.5 (2.7)	8.5 (2.6)
APACHE 2, points	24.8 (7.3)	27.5 (7.4)	23.7 (7.0)
Laboratory values, mean (SD)			
WBCC, /μL	17.8 (12.2)	18.1 (16.3)	17.7 (10.1)
Lactate, mmol/L	3.6 (3.3)	4.2 (3.5)	3.4 (3.2)
PCT, ng/mL	24.2 (55.1)	32.4 (66.8)	21.0 (49.3)
CRP, mg/L	203.1 (115.4)	212.4 (115.0)	199.4 (115.5)
Causes of ICU admission, *n* (%)			
Sepsis	135 (20)	27 (14)	108 (22)
Septic shock	555 (80)	169 (86)	386 (78)
Scr before study inclusion, mean (SD)	143.7 (95.3)	162.5 (100.1)	136.2 (92.4)
Renal function at study inclusion, mean (SD)			
Scr, μmol/L	149.2 (91.4)	174.5 (95.7)	136.8 (86.8)
Urea, mmol/L	11.4 (7.0)	13.9 (8.2)	10.2 (6.0)
eGFR, ml/min	60.0 (33.0)	53.0 (32.1)	62.8 (33.0)
Day of max. Scr, mean (SD)	2.7 (4.9)	6.4 (6.0)	1.1 (3.3)
Dynamic and functional biomarkers of interest at study inclusion			
kineGFR, mL/min*, mean (SD)	59.8 (30,4)	53.5 (27.3)	62.4 (31.4)
H3.1, ng/mL, geom mean (geom SD)	732.4 (3.7)	1,141.7 (4.0)	614.8 (3.4)
MR-ProADM, nmol/L, mean (SD)	6.3 (4.9)	8.8 (5.8)	5.4 (4.1)

Demographics, comorbidities and disease severity

*AKD* acute kidney disease, *DM* diabetes mellitus, *SOFA* Sequential Organ Failure Assessment Score, *APACHE* Acute Physiology And Chronic Health Evaluation, *WBCC* white blood cell count, *PCT* procalcitonin, *CRP* C-reactive protein, *Scr* Serum creatinine, *GFR* glomerular filtration rate, *KDIGO* Kidney Disease Improving Global Outcomes, *RRT* renal replacement therapy, kineGFR* d 0 to d minus 1, *H3.1* Histones H3.1, *MR-ProADM* midregional pro-adrenomedullin

Data are presented as means (SD), n (%) or geom. mean (geom. SD) for H3.1. Baseline Serum creatinine defined as the lowest value within 24 h prior to inclusion

### Longitudinal biomarker trajectories

Longitudinal analyses included only patients who were not receiving RRT at the respective study time points.

**Fig. 2:**
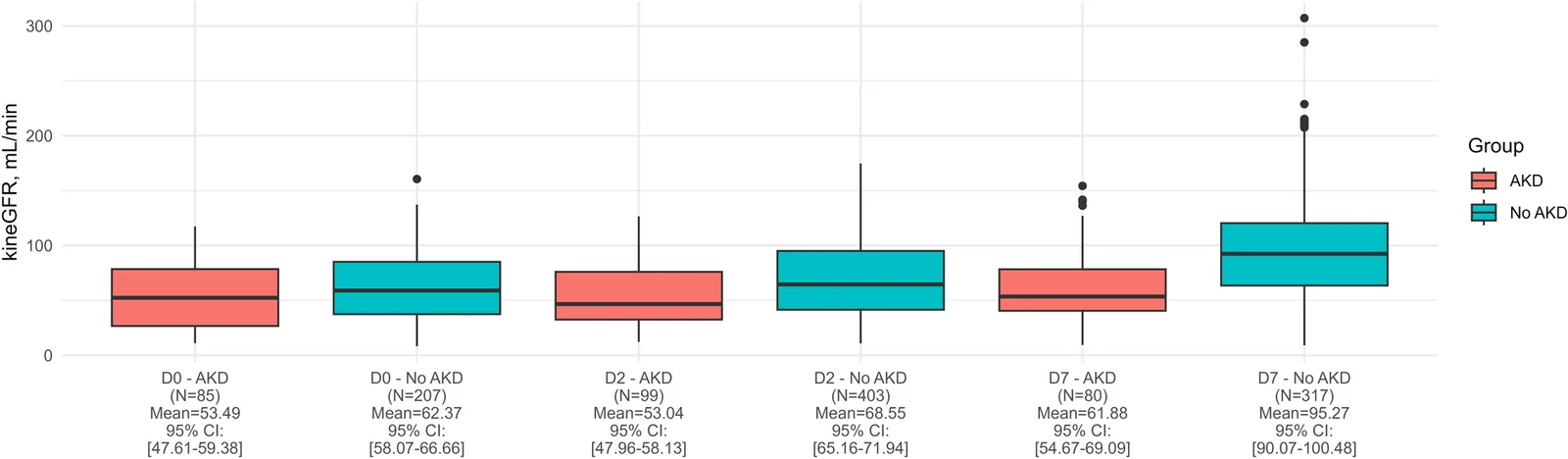
Kinetic eGFR over time. Boxplots of kinetic eGFR concentrations at day 0 (D0), day 2 (D2), and day 7 (D7) in patients with and without AKD

Median kinetic eGFR increased steadily from day 0 to day 7 in patients without AKD (59–92 mL/min), whereas it remained persistently reduced in patients developing AKD (52–53 mL/min), indicating sustained impairment of renal function (Supplementary Table S1, Fig. 2).

**Fig. 3:**
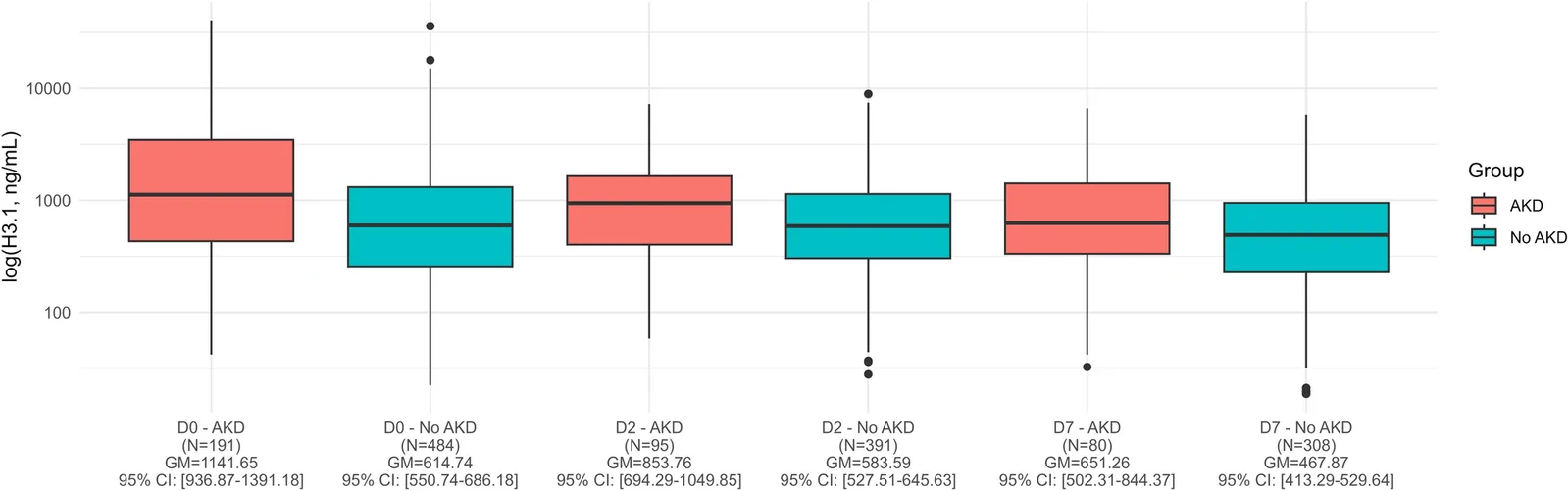
Histone H3.1 trajectories over time. Boxplots of circulating histone H3.1 concentrations at day 0 (D0), day 2 (D2), and day 7 (D7) in patients with and without AKD

**Fig. 4:**
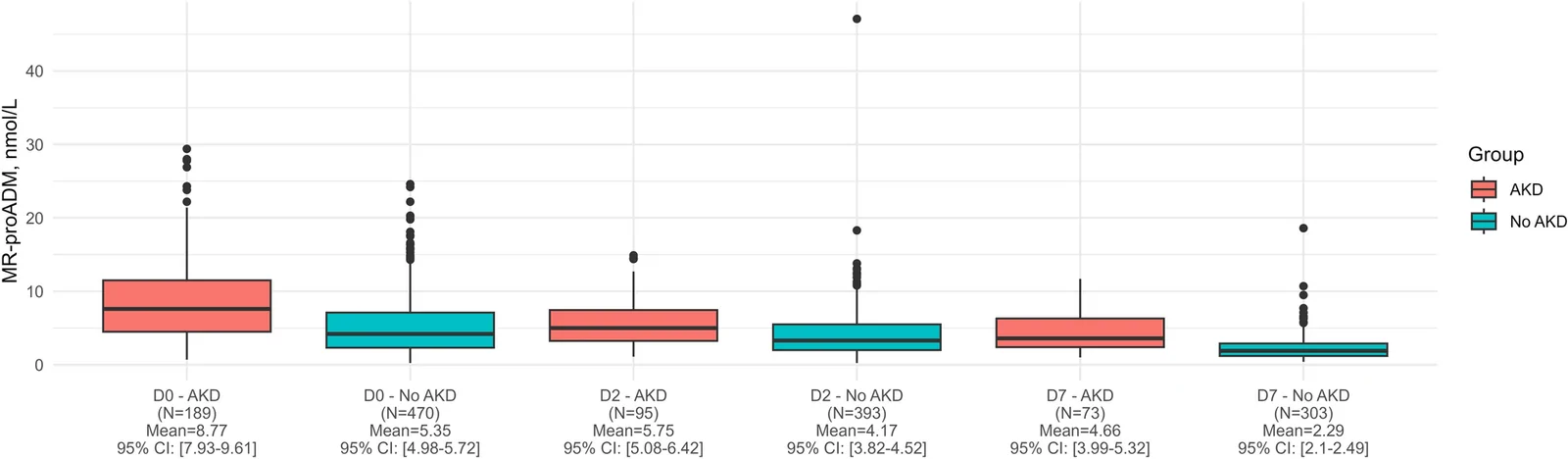
MR-proADM trajectories over time. Boxplots of MR-proADM concentrations at day 0 (D0), day 2 (D2), and day 7 (D7) in patients with and without AKD

H3.1 concentrations decreased during follow-up in both groups but remained consistently higher among patients with AKD throughout the observation period (Fig. 3, Supplementary Table S1). Likewise, MR-proADM concentrations declined over time but remained substantially elevated in patients who subsequently developed AKD compared with those without AKD (Fig. 4, Supplementary Table S1).

### Association with acute kidney disease

In univariable analyses, older age, male sex, higher non-renal SOFA score, lower baseline kinetic eGFR, and higher baseline concentrations of MR-proADM and H3.1 were associated with AKD (Table 2).

**Table 2:** Unadjusted odds ratios for acute kidney disease (AKD) at baseline; *n* = 690

Variable	OR	95%CI
Kinetic eGFR	0.990	0.981–0.999
MR-proADM	1.150	1.109–1.194
H3.1 (log)	1.454	1.272–1.667
Nonrenal SOFA	1.153	1.083–1.230
Female	0.630	0.434–0.904
age	1.015	1.002–1.029

*OR* odds ratio, *CI* confidence intervals, *Kinetic eGFR* kinetic estimated glomerular filtration rate, *MR-proADM* midregional proadrenomedullin, *SOFA* Sequential Organ Failure Assessment Score

Odds ratios represent the effect per one-unit increase in each predictor. Age is per year, non-renal SOFA per point, kinetic eGFR per 1 ml/min/1.73 m^2^, and MR-proADM per 1 nmol/L. For log-transformed H3.1, one-unit increase corresponds to an approximately 2.72-fold increase in the original scale

After adjustment for age, sex and non-renal SOFA score, only baseline MR-proADM remained independently associated with AKD (adjusted OR 1.17, 95% CI 1.08–1.28; *p* < 0.001). Neither baseline kinetic eGFR nor H3.1 retained independent significance.

At day 2, only changes in kinetic eGFR remained independently associated with subsequent AKD (adjusted OR 0.95, 95% CI 0.93–0.97; *p* < 0.001), whereas changes in MR-proADM and H3.1 were not independently associated with outcome. Similar findings were observed at day 7, where kinetic eGFR remained the only independent predictor of AKD (adjusted OR 0.96, 95% CI 0.94–0.98; *p* < 0.001) (Table 3).

**Table 3:** Adjusted logistic regression model for acute kidney disease, *n* = 690

Variable	Adjusted OR	95%CI	p-value
*Baseline* *N* = *259*			
Kinetic eGFR	1.003	0.991–1.015	0.669
MR-proADM	1.171	1.081–1.279	< 0.001
H3.1 (log)	1.210	0.940–1.566	0.143
Nonrenal SOFA	1.119	0.992–1.267	0.070
Female sex	0.462	0.228–0.894	0.026
Age	1.014	0.990–1.039	0.264
*Day 2 (∆ values)* *N* = *201*			
Kinetic eGFR	0.950	0.926–0.972	< 0.001
MR-proADM	0.894	0.747–1.017	0.173
H3.1 (log)	1.000	0.9997–1.0001	0.598
Nonrenal SOFA	1.100	0.943–1.286	0.229
Female	0.501	0.208–1.119	0.104
Age	1.006	0.982–1.033	0.627
*Day 7 (∆ values)* *N* = *164*			
Kinetic eGFR	0.960	0.941–0.977	< 0.001
MR-proADM	0.995	0.880–1.131	0.941
H3.1 (log)	1.000	0.9996–1.0001	0.382
Nonrenal SOFA	1.065	0.880–1.286	0.515
Female	0.464	0.163–1.192	0.126
Age	1.008	0.977–1.041	0.636

*OR* odds ratio, *CI* confidence intervals, *Kinetic eGFR* kinetic estimated glomerular filtration rate, *MR-proADM* midregional proadrenomedullin, *SOFA* Sequential Organ Failure Assessment Score

Odds ratios represent the effect per one-unit increase in each predictor. Age is per year, non-renal SOFA per point, kinetic eGFR per 1 ml/min/1.73 m^2^, and MR-proADM per 1 nmol/L. For log-transformed H3.1, one-unit increase corresponds to an approximately 2.72-fold increase in the original scale

### Predictive performance

**Fig. 5:**
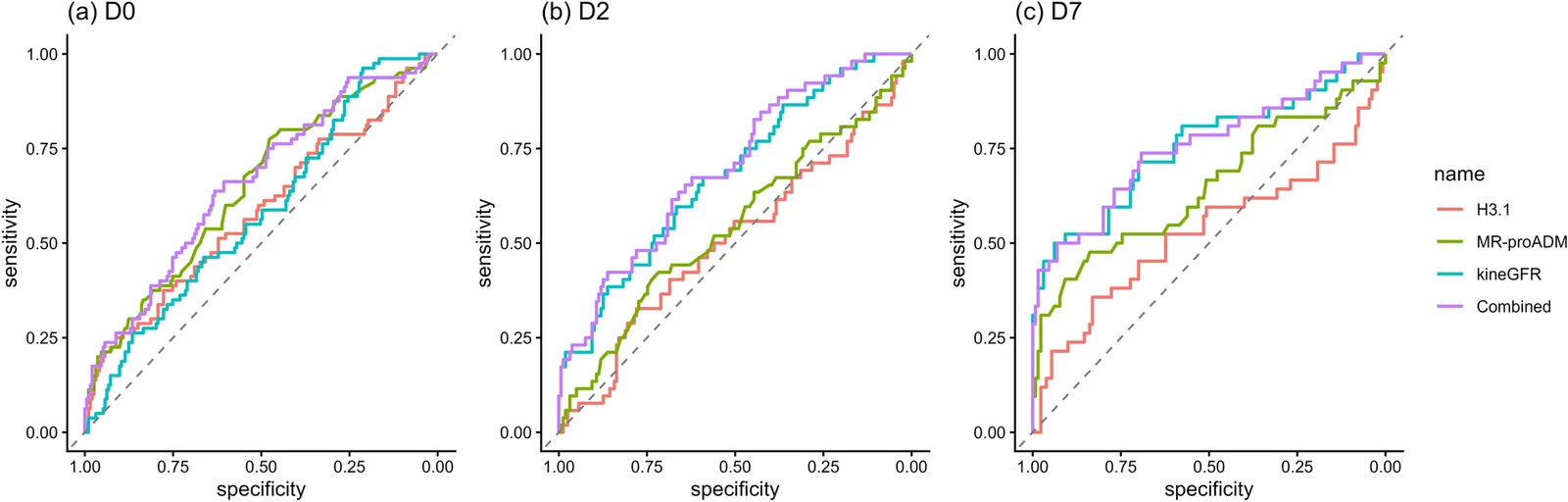
ROC-curves of biomarker and model-based prediction of AKD at **a** d0, **b** d2, **c** d7

Discriminative performance to predict the endpoint of AKD using combined biomarker models improved over time (Table 4, Fig. 5 a–c). At baseline, the combined model yielded an AUC of 0.65 (95% CI 0.59–0.73), only marginally exceeding MR-proADM alone in the point-estimates (AUC 0.64; 95% CI 0.58–0.72), whereas kinetic eGFR and H3.1 showed limited discrimination (Table 4, Fig. 5 a).

**Table 4:** Diagnostic performance of biomarkers for prediction of AKD

Timepoint	Biomarker	AUC (95% CI)	Treshold	Sensitivity	Specificity	NPV	PPV	Pos. DLR	Neg. DLR
D0 *N* = 273	kineGFR	0.58 (0.51–0.65)	94.61 [mL/min]	0.96	0.21	0.93	0.34	1.22	0.18
	H3.1	0.58 (0.51–0.66)	2534.48 [ng/mL]	0.25	0.91	0.74	0.54	2.84	0.82
	MR-proADM	0.64 (0.58–0.72)	3.85 [nmol/L]	0.78	0.48	0.84	0.38	1.48	0.47
	Combined	0.65 (0.59–0.73)	0.26	0.64	0.63	0.81	0.42	1.73	0.57
D2 *N* = 211	Diff. kineGFR	0.68 (0.59–0.76)	0.12 [mL/min]	0.67	0.59	0.85	0.35	1.65	0.55
	Diff. H3.1	0.51 (0.41–0.60)	396.23 [ng/mL]	0.33	0.78	0.78	0.33	1.49	0.86
	Diff. MR-proADM	0.54 (0.45–0.64)	0.25 [nmol/L]	0.42	0.72	0.79	0.33	1.49	0.80
	Combined	0.70 (0.62–0.78)	0.24	0.67	0.62	0.85	0.37	1.78	0.53
D7 *N* = 172	Diff. kineGFR	0.76 (0.66–0.85)	-8.03 [mL/min]	0.50	0.94	0.85	0.72	8.13	0.53
	Diff. H3.1	0.53 (0.41–0.64)	321.68 [ng/mL]	0.36	0.83	0.80	0.41	2.11	0.77
	Diff. MR-proADM	0.65 (0.54–0.76)	-0.2 [nmol/L]	0.48	0.84	0.83	0.49	2.95	0.62
	Combined	0.76 (0.66–0.85)	0.40	0.50	0.93	0.85	0.70	7.22	0.54

Thresholds were determined using the Youden index. PPV and NPV depend on cohort prevalence

*AUC* area under the ROC curve, *NPV* negative predictive value, *PPV* positive predictive value, *pos.* positive; *neg.* negative, *DLR* diagnostic likelihood ratio, *kineGFR* kinetic estimated glomerular filtration rate, *MR-proADM* midregional proadrenomedullin, *Diff.* difference

For odds ratios, 95%-CI, regression coefficients and intercept of the combined models see Table S2

At day 2, predictive performance increased in the combined model (AUC 0.70; 95% CI 0.62–0.78), with kinetic eGFR providing the strongest discrimination among the individual biomarkers (AUC 0.68; 95% CI 0.59–0.76) (Table 4, Fig. 5 b).

By day 7, the combined model achieved an AUC of 0.76 (95% CI 0.66–0.85), which was comparable to kinetic eGFR alone (AUC 0.76; 95% CI 0.66–0.85), while MR-proADM showed moderate discrimination (AUC 0.65; 95% CI 0.54–0.76) and H3.1 demonstrated poor performance (AUC 0.53; 95% CI 0.41–0.64) (Table 4, Fig. 5 c).

Overall, dynamic changes in kinetic eGFR consistently outperformed the circulating biomarkers after baseline, whereas H3.1 provided little incremental predictive information. The combined model consistently had (one of) the highest AUC values. However, based on the 95% confidence intervals, it cannot be concluded that it is superior (Supplementary Table S2).

### Calibration

**Fig. 6:**
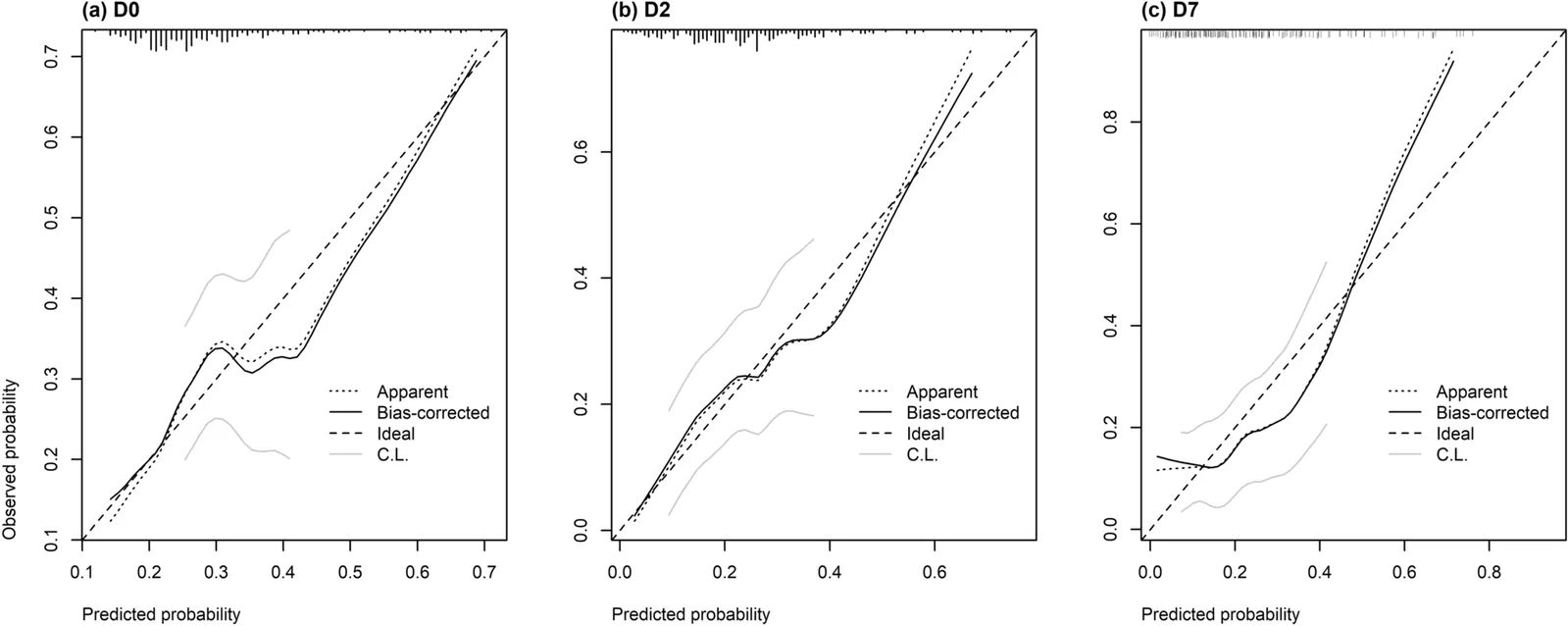
Bootstrap-corrected calibration plots for AKD prediction using biomarker-based and combined models at **a** D0, **b** D2, and **c** D7

Across all time points (D0, D2, and D7), the combined models demonstrated overall good calibration performance. Calibration intercepts were close to zero and calibration slopes were close to, although consistently below, one, indicating only minor systematic overestimation of risk and limited overfitting. The Brier score showed a gradual improvement over time, decreasing from 0.190 at D0 to 0.163 at D2 and 0.142 at D7, reflecting improved overall predictive accuracy at later time points (Table 5). Visual inspection of the bootstrap-corrected calibration plots confirmed these findings, showing generally good agreement between predicted and observed probabilities across most of the risk spectrum. At D0 and D2 minor local deviations were observed in the mid-risk range. At D7, the calibration curve suggested more pronounced non-linear miscalibration, with overestimation of risk in the lower-to-mid probability range and underestimation at higher predicted probabilities, indicating a modest deterioration in calibration across the risk spectrum, consistent with the slightly lower bootstrap optimism-corrected calibration slope, despite overall good global calibration metrics (Fig. 6).

**Table 5:** Calibration of combined models; *n* = 690

Timepoint	Calibration intercept	Calibration slope	Brier score
D0 *N* = 273	−0.051	0.936	0.190
D2 *N* = 211	−0.035	0.952	0.163
D7 *N* = 172	−0.079	0.882	0.142

*D* day

### Clinical utility assessment using decision curve analysis

**Fig. 7:**
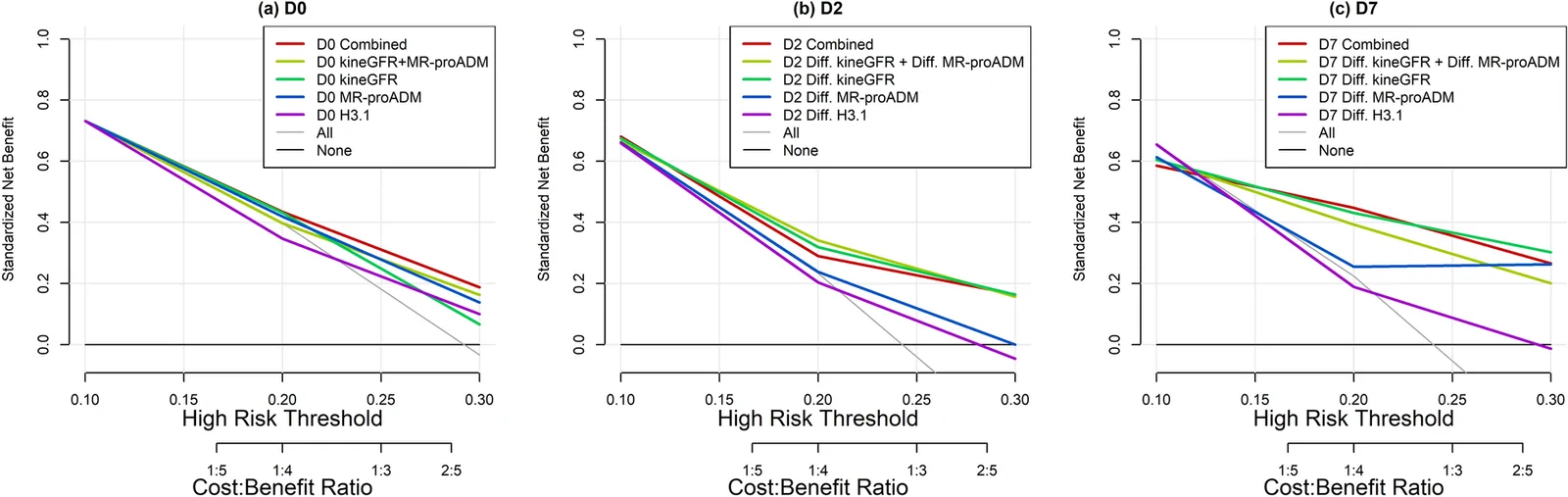
Decision curves of biomarker and model-based prediction of AKD at **a** d0, **b** d2, **c** d7

Decision curve analysis (DCA) demonstrated that the combined biomarker model provided a clinical net benefit across most evaluated threshold probabilities compared with individual biomarkers and default strategies (“treat all” or “treat none”) (Fig. 7).

By day 2, the combined model reaches the highest net benefit within parts of the threshold range, while kinetic eGFR changes show a comparable performance. The remaining biomarkers contribute less consistently to clinical net benefit.

At day 7, the combined model remains competitive and typically ranks among the better-performing approaches, although it is not uniformly superior. Overall, these findings suggest that the combined model is capable of supporting clinical decision-making.

Due to the estimates obtained through cross-validation, the exact rankings in these figures should only be understood as trends.

### Secondary endpoints

#### Mortality

The mean follow-up time of the cohort was 69 ± 33 days, reflecting the duration of available SCr measurements and clinical outcome assessment. Follow-up was censored at death or last available laboratory measurement.

Patients with AKD had substantially higher mortality compared with those without AKD. Twenty-eight-day mortality was 33% in patients with AKD versus 15% in those without AKD. Ninety-day mortality was 55% in patients with AKD compared with 24% in those without AKD (see Table 6). This results in a risk ratio of 2.3 (95%-CI 1.9–2.9) for 90-day mortality.

**Table 6:** 28- and 90-day mortality

variable	Total *N* = 690	AKD *N* = 196	No AKD *N* = 494
28-day mortality, No. (%)	137 (19.9)	64 (33)	73 (15)
Unknown	7	0	7
90-day mortality, No. (%)	217 (31.5)	105 (55)	112 (24)
Unknown	26	5	21

*AKD* acute kidney disease

Percentages were calculated excluding patients with missing follow-up

Evaluation of biomarker performance for prediction of 90-day mortality demonstrated only modest discrimination. Baseline MR-proADM showed the highest performance (AUC 0.68), whereas H3.1 and the combined model showed no meaningful improvement. At days 2 and 7, neither biomarker changes nor the combined model reliably predicted mortality (Supplementary Table S3, Supplementary Figure S3).

#### Renal recovery (after AKD)

Among patients with AKD, complete or partial renal recovery occurred in 62 patients (32%), whereas 128 patients (67%) experienced no recovery or died before recovery could be documented (Table 7). Baseline kinetic eGFR tended to be lower and MR-proADM concentrations higher in patients without recovery, while H3.1 showed no consistent association.

**Table 7:** Renal recovery outcomes in patients with acute kidney disease, *n* = 196

Recovery	Total *N* = 196	Previous AKI *N* = 167	No previous AKI *N* = 29
Compl. recovery, No. (%)	25 (13)	19 (12)	6 (21)
RRT	15	13	2
No RRT	10	6	4
Good Part. recovery, No. (%)	18 (10)	17 (11)	1 (3)
RRT	12	12	0
No RRT	6	5	1
Lim. Part. recovery, No. (%)	19 (10)	18 (11)	1 (3)
RRT	11	11	0
No RRT	8	7	1
No Recovery / Non-Survivor, No. (%)	128 (67)	107 (66)	21 (72)
RRT/Death	127 (22/105)	107 (19/88)	20 (3/17)
No RRT	1	0	1
Unknown, No	6	6	0

*AKD* acute kidney disease, *compl.* complete, *part.* partial, *RRT* renal replacement therapy

#### Biomarker trajectories and predictive performance of 90-day mortality and recovery

Supplementary Figures S1 and S2 show biomarker trajectories in survivors versus non-survivors, whereas Supplementary Figures S4–S6 depict trajectories according to renal recovery status. Overall, biomarker dynamics demonstrated substantial overlap between groups across time points.

For prediction of 90-day mortality, ROC analysis (Supplementary Figure S3 and Supplementary Table S3) demonstrated only modest discriminatory ability at baseline, with AUCs of 0.57 for H3.1 and 0.68 for both MR-proADM and the combined model. Discriminatory performance further declined at later time points (Day 2 and Day 7), with AUCs approximating 0.5, indicating limited predictive value.

Prediction of renal recovery was inconsistent across time points. At baseline, only the combined model showed moderate discrimination (AUC 0.66). At day 2, kinetic eGFR (AUC 0.78) and the combined model (AUC 0.77) demonstrated the best predictive performance. In contrast, analyses at day 7 showed poor discrimination for all biomarkers and should be interpreted cautiously because of the limited number of evaluable patients (*n* = 41) (Supplementary Table S4, Supplementary Figures S4–S7).

## Discussion

In this secondary analysis of a large multicentre randomized controlled trial, we evaluated the predictive performance of kinetic eGFR, MR-proADM, H3.1 nucleosomes, and a combined multimarker model for predicting acute kidney disease (AKD), defined according to the contemporary ADQI criteria, as well as 90-day mortality and renal recovery in patients with sepsis. The main findings are as follows: (1) MR-proADM showed the strongest discrimination among the individual biomarkers at study inclusion, whereas dynamic changes in kinetic eGFR provided the most consistent predictive information during follow-up. (2) Although combining biomarkers modestly (if at all) improved discrimination at baseline compared with the individual markers, this advantage diminished over time and largely reflected the contribution of kinetic eGFR. (3) Decision curve analysis likewise suggested only modest additional clinical utility of the combined model, while calibration analyses demonstrated acceptable overall model performance and finally. (4) AKD was strongly associated with increased mortality and low rates of renal recovery.

The rationale for combining biomarkers capturing different biological pathways—cellular injury (H3.1), endothelial dysfunction (MR-proADM), and renal function (kinetic eGFR)—is well supported by the multifactorial pathophysiology of sepsis-associated AKI [1, 18–20]. Prior studies have shown that no single biomarker sufficiently captures this complexity, and multimarker strategies may improve risk stratification [21, 22].

The predictive performance of MR-proADM at baseline is consistent with its established role as a marker of endothelial dysfunction and microvascular injury, both of which are central mechanisms in sepsis-associated kidney injury [23–26]. Elevated MR-proADM concentrations have repeatedly been associated with organ dysfunction, disease severity, and adverse outcomes in patients with sepsis [25, 27]. Accordingly, our findings suggest that endothelial injury is already present at the onset of sepsis, before measurable deterioration of renal function becomes apparent, which likely explains the moderate baseline discrimination of MR-proADM for subsequent AKD. However, its predictive value diminished over time and was not clearly superior to the combined model, indicating that MR-proADM primarily reflects early systemic disease severity rather than ongoing kidney-specific pathophysiological processes. These findings underscore that, although endothelial dysfunction is biologically relevant in the development of sepsis-associated AKD, endothelial biomarkers alone provide only limited incremental value for predicting persistent renal dysfunction.

In contrast, kinetic eGFR became the strongest predictor during follow-up. Unlike conventional eGFR equations, kinetic eGFR accounts for the dynamic changes in SCr that occur during non-steady-state conditions and therefore better reflects evolving renal function during critical illness [12, 13]. At both Day 2 and Day 7, changes in kinetic eGFR consistently outperformed the circulating biomarkers and largely explained the predictive performance of the combined model. Kinetic eGFR, by incorporating dynamic changes in SCr and accounting for non-steady-state kinetics, reflects real-time renal function and has been shown to outperform static SCr-based estimates in such conditions [12, 28], supporting previous findings that dynamic functional assessment may provide greater prognostic value than isolated injury biomarker measurements once renal dysfunction has evolved.

Circulating H3.1 nucleosomes demonstrated limited predictive performance throughout the study. Although extracellular histones are increasingly recognized as mediators of inflammation, endothelial injury, and immunothrombosis in sepsis [29, 30], the present data suggest that circulating H3.1 concentrations provide little incremental information for predicting subsequent AKD beyond routinely available clinical and functional measures. Several explanations are possible. Histone release reflects systemic cell injury and neutrophil extracellular trap formation rather than kidney-specific damage, resulting in considerable overlap between patients with and without AKD. Furthermore, the temporal kinetics of histone release during sepsis may not coincide with the time window relevant for predicting persistent kidney dysfunction. However, elevated levels of H3.1 were recently found to be associated with an increase mortality and necessity of RRT (Neumann et al., accepted for CCM, 2026). Nonetheless, in our study, patients with RRT were excluded for evaluation of prediction models, as hemofiltration alters the levels of biomarkers in an unpredictive manner. Our findings are still in line with studies showing that markers of generalized cell damage often add limited incremental value when more direct functional measures are available [22].

For secondary endpoints, biomarker performance was modest for mortality prediction and inconsistent for renal recovery. While kinetic eGFR and the combined model showed some signal at day 2 for recovery prediction, this was not sustained across time points. These findings likely reflect both biological and methodological factors: On the one hand, from a pathophysiological perspective, renal recovery is influenced by a complex interplay of injury severity, repair mechanisms, and systemic factors, which may not be adequately captured by the selected biomarkers alone. On the other hand, methodologically, the small sample size—particularly at later time points—introduces substantial uncertainty. This limitation is reflected in the wide variability of ROC estimates and underscores the need for cautious interpretation.

Overall, our findings suggest that dynamic assessment of renal function using kinetic eGFR remains the most informative predictor of AKD after the initial presentation of sepsis. While MR-proADM provides useful baseline prognostic information, and multimarker models demonstrate acceptable calibration and modest clinical utility, the incremental benefit beyond kinetic eGFR alone appears limited. Future studies should focus on externally validating these findings and on identifying biomarker combinations that provide clinically meaningful improvements over routine functional assessment.

## Strength and limitations

This study has several strengths. It is based on a large, well-characterized, multicentre cohort of critically ill patients with sepsis, enhancing the robustness and generalizability of the findings within ICU populations. The use of prospectively collected data and standardized definitions for AKI and AKD strengthens internal validity. Importantly, we evaluated biomarkers representing complementary pathophysiological domains—cellular injury, endothelial dysfunction, and dynamic renal function—allowing a comprehensive assessment of their individual and combined predictive value. The longitudinal design with repeated measurements enabled analysis of temporal biomarker trajectories and their association with outcomes. Another important strength of the present study is its comprehensive evaluation of model performance beyond discrimination, incorporating both calibration and decision curve analysis with cross-validation to better assess potential clinical utility. While calibration indicated generally acceptable agreement between predicted and observed risks across time points, some local miscalibration was observed at Day 7. Decision curve analysis suggested only modest and inconsistent clinical benefit of the combined model across selected threshold probabilities, with no clear or sustained advantage over kinetic eGFR alone. These findings highlight that improvements in discrimination do not necessarily translate into meaningful gains in clinical decision-making and support a cautious interpretation of the incremental value of multimarker prediction approaches. Beyond AKD prediction, neither H3.1 nor MR-proADM substantially improved prediction of 90-day mortality or renal recovery. Baseline MR-proADM demonstrated only moderate discrimination for mortality, whereas predictive performance of all biomarkers declined during follow-up. Similarly, prediction of renal recovery remained inconsistent across all evaluated time points. These observations likely reflect the multifactorial determinants of long-term outcomes in septic patients, in which kidney-specific biomarkers capture only one component of an increasingly complex pathophysiological process.

Several limitations should be considered. First, this represents a secondary analysis of a randomized controlled trial that was not originally designed to evaluate biomarker-guided prediction of AKD. Second, a well-known risk factor for the development of AKD, chronic-kidney disease (CKD), was not available in the dataset and could therefore not be implemented in the analyses. Although internal validation using cross-validation and bootstrap calibration was performed, external validation in independent sepsis cohorts remains necessary before clinical implementation can be considered. Third, biomarker measurements were available only at predefined study time points, potentially limiting characterization of individual biomarker trajectories. Finally, despite the inclusion of complementary biomarkers reflecting functional impairment, endothelial injury, and cell damage, the overall incremental predictive value beyond dynamic assessment of renal function remained modest, indicating that additional biomarkers or multimodal prediction approaches may be required to achieve clinically relevant improvements.

## Conclusions

In this multicentre secondary analysis of a randomized controlled trial in patients with sepsis, we demonstrated that dynamic assessment of renal function using kinetic eGFR provides the most robust and consistent prognostic information for the development of AKD during critical illness. Although MR-proADM showed useful early discriminatory ability and multimarker approaches modestly improved baseline performance, these advantages were not sustained over time and did not translate into clear or consistent gains in clinical utility. Circulating H3.1 nucleosomes contributed little additional predictive value, and overall improvements in discrimination did not meaningfully enhance decision-making as assessed by calibration and decision curve analysis. Together, these findings highlight the limited incremental benefit of combining current circulating biomarkers with functional measures and underscore the importance of dynamic renal function assessment in sepsis-associated kidney disease. Future research should focus on external validation and on identifying novel biomarker or multimodal strategies that can meaningfully improve prediction beyond kinetic assessment of kidney function alone.

## Supplementary Information

Supplementary Material 1.

## Data Availability

No datasets were generated or analysed during the current study.

## Abbreviations

AUC: Area under the curve
AKD: Acute kidney disease
AKI: Acute kidney injury
CI: Confidence intervals
CKD: Chronic kidney disease
CRP: C-reactive protein
DCA: Decision curve analysis
eGFR: Estimated glomerular filtration rate
ICU: Intensive care unit
MR-proADM: Midregional proadrenomedullin
OR: Odds ratio
PCT: Procalcitonin
ROC: Receiver operating curve
RRT: Renal replacement therapy
SA-AKI: Sepsis-associated acute kidney injury
SCr: Serum creatinine
SOFA: Sequential Organ Failure Assessment Score
