# Supplementary material for: Predictive performance of kinetic eGFR, midregional proadrenomedullin, and H3.1 nucleosomes for acute kidney disease in sepsis: a secondary analysis of a large multicentre randomized controlled trial
Source: J Intensive Care. 2026 Aug 19;14:62. doi: 10.1186/s40560-026-00926-y (PMC13488215; doi:10.1186/s40560-026-00926-y)
Supplement: Supplementary file 1 [file 40560_2026_926_MOESM1_ESM.docx]

**Supplement**

**Predictive performance of kinetic eGFR, midregional Proadrenomedullin, and H3.1 nucleosomes for acute kidney disease in sepsis: A secondary analysis of a large multicentre randomized controlled trial**

Caroline Neumann¹, Frank Bloos¹², Philipp Franken³, Thomas Lehmann³, Holger Bogatsch⁴,

Johannes Ruhe⁵ and the Sepnet Critical Care Trials Group

## **Measurement of biomarkers and calculation of kinetic eGFR**

**H3.1 nucleosome measurement (Nu.Q® H3.1 immunoassay)**

H3.1 nucleosomes were measured in frozen Citrate plasma samples using the Nu.Q H3.1® Immunoassay (Belgian Volition SRL, Isnes, Belgium) according to the manufacturer’s instruction [1]. This sandwich immunoassay is based on magnetic beads and chemiluminescence technology and is performed on the IDS-i10 automated immunoanalyzer system (Immunodiagnostic Systems Ltd, UK). 50 μL of citrate plasma are incubated with acridinium ester labeled anti-nucleosome antibody. Then, magnetic particle beads, coated with the monoclonal anti-histone modification capture antibody are added. Finally, after a wash cycle, trigger solutions are added, and the light emitted by the acridinium ester is measured by the luminometer system. The results are expressed in relative light unit (RLU) and the concentrations are extrapolated using a four-parameter logistic regression of a reference standard curve. According to the manufacturer’s instructions for use (IFU), the assay has a limit of detection (LoD) of 2 ng/mL and a limit of quantification (LoQ) of 3 ng/mL. The reportable range is 22.2–1217.4 ng/mL for frozen samples. Reported intra-assay coefficients of variation (CVs) range from 1.6% to 2.6%, and inter-assay CVs range from 5.3% to 7.5% (frozen samples). In addition, independent analytical validation has demonstrated robust assay performance, including an LoD of approximately 0.93 ng/mL, LoQ of approximately 2.3 ng/mL, and validated linearity across a broad measurement range, further supporting the analytical reliability of the assay [1].

**Midregional-proADM measurement**

Midregional-proADM was measured in EDTA plasma using commercially available automated immunofluorescent assays (MR-proADM™ KRYPTOR™ and PCT™ sensitive KRYPTOR™, B·R·A·H·M·S GmbH, Hennigsdorf, Germany) [2]. The assay is calibrated against a freeze-dried synthetic MR-proADM reference preparation, as no international reference standard is available. The analytical measurement range is linear from 0.21 to 100 nmol/L, with a reportable range from the limit of detection (LoD) to 10 nmol/L without dilution and up to 100 nmol/L with automatic dilution. The LoD and limit of quantitation (LoQ) are 0.09 nmol/L and 0.21 nmol/L, respectively.

Assay precision was evaluated according to CLSI guidelines (EP05-A3). Intra-assay (repeatability) coefficients of variation (CVs) ranged from 1.0% to 7.6%, while inter-assay (within-laboratory) CVs ranged from 4.9% to 15.2%, depending on concentration. Between-run reproducibility across instruments showed CVs between 1.6% and 6.8%. The assay demonstrated no clinically relevant interference from endogenous substances (e.g., hemoglobin, bilirubin, triglycerides, rheumatoid factor) or common medications and cross-reacting biomarkers at tested concentrations. No high-dose hook effect was observed up to 100 nmol/L. In healthy individuals, the 97.5th percentile for MR-proADM is 0.55 nmol/L.

**Kinetic eGFR estimation**

Kinetic eGFR was estimated using the modified Chen formula [3], where baseline Serum creatinine (SCr) is the baseline value 24 h preceding study inclusion. SCr (H24) is the value of the day to evaluate kinetic eGFR (KeGFR) and SCr(H0) was the day before. KeGFR is a modified calculation of kidney function that accounts for rapid changes in serum creatinine levels. Unlike conventional eGFR equations, which assume steady-state creatinine concentrations, kineGFR incorporates the rate of change in creatinine to better estimate glomerular filtration during non-steady-state conditions.

**Supplementary Table S1: Dynamic and functional biomarkers of interest at baseline, day 2 and day 7 in patients with and without AKD**

| **variable** | **n** | **Total**  **N=690** | **AKD**  **N=196** | **No AKD**  **N=494** |
| --- | --- | --- | --- | --- |
| **kineGFR**, mL/min*  Baseline, median (Q1, Q3) | 292 | 57 (34, 83) | 52 (27, 78) | 59 (37, 86) |
| Day 1 | 266 | 56 (37, 83) | 48 (30, 73) | 58 (38, 87) |
| Day 2 | 502 | 61 (39, 90) | 47 (32, 76) | 65 (41, 95) |
| Day 3 | 477 | 69 (42, 97) | 52 (30, 76) | 75 (45, 101) |
| Day 4 | 447 | 75 (46, 103) | 58 (34, 80) | 82 (48, 107) |
| Day 5 | 436 | 81 (51, 107) | 58 (39, 88) | 86 (54, 113) |
| Day 6 | 427 | 82 (54, 112) | 57 (41, 83) | 88 (59, 117) |
| Day 7 | 397 | 82 (55, 116) | 53 (40, 79) | 92 (64, 120) |
| **H3.1**, ng/mL  Baseline, geom. Mean (geom. SD) | 675 | 732.4 (3.7) | 1,141.7 (4.0) | 614.7 (3.4) |
| Day 2 | 624 | 747.2 (3.0) | 1,145.5 (3.0) | 627.5 (2.9) |
| Day 7 | 536 | 591.7 (3.1) | 825.7 (3.0) | 493.4 (3.0) |
| **MR-proADM**, nmol/L  Baseline, median (Q1, Q3) | 659 | 5.0 (2.7, 8.6) | 7.6 (4.5, 11.5) | 4.2 (2.3, 7.1) |
| Day 2 | 659 | 4.7 (2.6, 7.8) | 7.4 (4.6, 10.9) | 3.7 (2.1, 6.4) |
| Day 7 | 542 | 2.7 (1.6, 5.0) | 6.0 (3.6, 9.0) | 2.0 (1.2, 3.1) |

**Abbreviations: AKD:** acute kidney disease; **GFR:** glomerular filtration rate; **kineGFR***: d 0 to d minus 1; **H3.1**: Histones H3.1; **MR- ProADM:** Midregional Pro-Adrenomedullin; Data are presented as medians (Q1, Q3) or geom. mean (geom. sd) for H3.1.

**Supplementary Table S2: combined models for acute kidney disease, n=690**

| **Variable** | **Estimate** | **Adjusted OR** | **95%CI** |
| --- | --- | --- | --- |
| *Baseline*  ***N=273*** |  |  |  |
| (Intercept) | -2.78 |  |  |
| Kinetic eGFR | 0.00 | 1.00 | 0.99 – 1.01 |
| MR-proADM | 0.12 | 1.13 | 1.05 – 1.22 |
| H3.1 (log) | 0.16 | 1.18 | 0.93 – 1.50 |
|  |  |  |  |
| *Day 2 (∆ values)*  ***N=211*** |  |  |  |
| (Intercept) | -1.22 |  |  |
| Kinetic eGFR | -0.05 | 0.95 | 0.93 – 0.97 |
| MR-proADM | -0.10 | 0.90 | 0.77 – 1.02 |
| H3.1 (log) | -0.00 | 1.00 | 1.00 – 1.00 |
|  |  |  |  |
| *Day 7 (∆ values)*  ***N=172*** |  |  |  |
| (Intercept) | -0.68 |  |  |
| Kinetic eGFR | -0.04 | 0.96 | 0.94 – 0.98 |
| MR-proADM | 0.01 | 1.01 | 0.90 – 1.15 |
| H3.1 (log) | 0.00 | 1.00 | 1.00 – 1.00 |

**Abbrev.: OR:** odds ratio, **CI:** confidence intervals, **kinetic eGFR:** kinetic estimated glomerular filtration rate**, MR-proADM:** midregional Proadrenomedullin; Odds ratios represent the effect per one-unit increase in each predictor. kinetic eGFR per 1 ml/min/1.73 m², and MR-proADM per 1 nmol/L. For log-transformed H3.1, one unit increase corresponds to an approximately 2.72-fold increase in the original scale.

**Supplementary Figure S1**: **Histone H3.1 trajectories over time.** Boxplots of circulating histone H3.1 concentrations at day 0 (D0), day 2 (D2), and day 7 (D7) in survivors and non-survivors.

**Supplementary Figure S2:** **MR-proADM trajectories over time.** Boxplots of MR-proADM concentrations at day 0 (D0), day 2 (D2), and day 7 (D7) in in survivors and non-survivors. **Abbrev.:** **MR-proADM:** Midregional Pro-Adrenomedullin

**Supplementary Figure S3: ROC-curves of biomarker and model-based prediction of 90-day mortality at D0, D2, and D7**

**Supplementary Figure S4**: **Histone H3.1 trajectories over time.** Boxplots of circulating histone H3.1 concentrations at day 0 (D0), day 2 (D2), and day 7 (D7) in patients with and without recovery.

**Supplementary Figure S5:** **MR-proADM trajectories over time.** Boxplots of MR-proADM concentrations at day 0 (D0), day 2 (D2), and day 7 (D7) in patients with and without recovery. **Abbrev.:** **MR-proADM:** Midregional Pro-Adrenomedullin

**Supplementary Figure S6:** **Kinetic eGFR from day 0 to day 7.** Boxplots of kinetic eGFR concentrations at day 0 (D0), day 2 (D2), and day 7 (D7) in patients with and without recovery. **Abbrev.: Kinetic eGFR:** kinetic estimated glomerular filtration rate

**Supplementary Table S3: Diagnostic performance of biomarkers for prediction of**

**90-day mortality**

| **Timepoint** | **biomarker** | **AUC**  **(95% CI)** | **treshold** | **sensitivity** | **specificity** | **NPV** | **PPV** | **Pos. DLR** | **Neg. DLR** |
| --- | --- | --- | --- | --- | --- | --- | --- | --- | --- |
| **D0**  **N=831** | H3.1 | 0.57  (0.53 – 0.61) | 1142.76  [ng/mL] | 0.40 | 0.73 | 0.75 | 0.38 | 1.47 | 0.82 |
|  | MR-proADM | 0.68  (0.64 – 0.72) | 5.45 [nmol/L] | 0.61 | 0.68 | 0.81 | 0.44 | 1.91 | 0.57 |
|  | Combined | 0.68  (0.64 – 0.72) | 0.28 | 0.60 | 0.69 | 0.81 | 0.45 | 1.95 | 0.58 |
| **D2**  **N=714** | Diff. H3.1 | 0.53  (0.48 – 0.58) | 1067.37  [ng/mL] | 0.16 | 0.92 | 0.73 | 0.44 | 1.94 | 0.91 |
|  | Diff. MR-proADM | 0.54  (0.49 – 0.59) | 0.65 [nmol/L] | 0.31 | 0.85 | 0.76 | 0.46 | 2.14 | 0.80 |
|  | Combined | 0.54  (0.49 – 0.59) | 0.29 | 0.47 | 0.67 | 0.76 | 0.37 | 1.45 | 0.78 |
| **D7**  **N=522** | Diff. H3.1 | 0.51  (0.45 – 0.56) | 1170.57  [ng/mL] | 0.17 | 0.91 | 0.70 | 0.47 | 1.92 | 0.91 |
|  | Diff. MR-proADM | 0.50  (0.45 – 0.56) | 0.2 [nmol/L] | 0.26 | 0.88 | 0.72 | 0.50 | 2.13 | 0.84 |
|  | Combined | 0.51  (0.45 – 0.56) | 0.33 | 0.26 | 0.88 | 0.72 | 0.49 | 2.08 | 0.85 |

Thresholds were determined using the Youden index. PPV and NPV depend on cohort prevalence. **Abbrev.: AUC**: area under the ROC curve, **NPV**: negative predictive value, **PPV**: positive predictive value; **DLR**: diagnostic likelihood ratio, **MR-proADM:** midregional Proadrenomedullin, **diff.:** difference

**Supplementary Figure S7: ROC-curves of biomarker and model-based prediction of no recovery vs recovery at (a) D0, (b) D2, (c) D7**

**Supplementary Table S4. Diagnostic performance of biomarkers for prediction of recovery vs no recovery**

| **Timepoint** | **biomarker** | **AUC**  **(95% CI)** | **Treshold** | **sensitivity** | **specificity** | **NPV** | **PPV** | **Pos. DLR** | **Neg. DLR** |
| --- | --- | --- | --- | --- | --- | --- | --- | --- | --- |
| **D0**  **N=79** | kineGFR | 0.37  (0.23 – 0.51) | 18.00  [mL/min] | 0.14 | 0.96 | 0.66 | 0.67 | 3.45 | 0.90 |
|  | H3.1 | 0.52  (0.38 – 0.65) | 3415.21  [ng/mL] | 0.28 | 0.86 | 0.67 | 0.53 | 1.97 | 0.84 |
|  | MR-proADM | 0.43  (0.30 – 0.56) | 4.05  [nmol/L] | 0.72 | 0.28 | 0.64 | 0.37 | 1.01 | 0.99 |
|  | Combined | 0.66  (0.52 – 0.79) | 0.45 | 0.59 | 0.82 | 0.77 | 0.65 | 3.26 | 0.50 |
| **D2**  **N=51** | Diff. kineGFR | 0.78  (0.65 – 0.90) | -2.45  [mL/min] | 0.85 | 0.65 | 0.87 | 0.61 | 2.40 | 0.23 |
|  | Diff. H3.1 | 0.66  (0.50 – 0.82) | 60.66  [ng/mL] | 0.75 | 0.61 | 0.79 | 0.56 | 1.94 | 0.41 |
|  | Diff. MR-proADM | 0.62  (0.47 – 0.78) | -0.35  [nmol/L] | 0.80 | 0.48 | 0.79 | 0.50 | 1.55 | 0.41 |
|  | Combined | 0.77  (0.65 – 0.90) | 0.25 | 0.95 | 0.55 | 0.94 | 0.58 | 2.10 | 0.09 |
| **D7**  **N=41** | Diff. kineGFR | 0.58  (0.39 – 0.78) | -34.11  [mL/min] | 0.35 | 1.00 | 0.69 | 1.00 | Inf | 0.65 |
|  | Diff. H3.1 | 0.63  (0.43 – 0.83) | 506.23  [ng/mL] | 0.47 | 0.92 | 0.71 | 0.80 | 5.65 | 0.58 |
|  | Diff. MR-proADM | 0.49  (0.31 – 0.67) | 1.55  [nmol/L] | 0.29 | 0.79 | 0.61 | 0.50 | 1.41 | 0.89 |
|  | Combined | 0.57  (0.38 – 0.76) | 0.43 | 0.53 | 0.67 | 0.67 | 0.53 | 1.59 | 0.71 |

**no recovery vs recovery**

Thresholds were determined using the Youden index. PPV and NPV depend on cohort prevalence. **Abbrev.: AUC**: area under the ROC curve, **NPV**: negative predictive value, **PPV**: positive predictive value; **DLR**: diagnostic likelihood ratio

Due to the limited number of evaluable patients at Day 7 (n = 41), the Recovery-ROC analyses are subject to considerable uncertainty and should be interpreted with caution.

1.	Wargnies, M., et al., *Quantification of H3.1-nucleosomes using a chemiluminescent immunoassay: A reliable method for neutrophil extracellular trap detection.* PLoS One, 2025. **20**(8): p. e0329352.

2.	Caruhel, P., et al., *Homogeneous time-resolved fluoroimmunoassay for the measurement of midregional proadrenomedullin in plasma on the fully automated system B.R.A.H.M.S KRYPTOR.* Clin Biochem, 2009. **42**(7-8): p. 725-8.

3.	Dewitte, A., et al., *Kinetic eGFR and Novel AKI Biomarkers to Predict Renal Recovery.* Clin J Am Soc Nephrol, 2015. **10**(11): p. 1900-10.
